# CSF, Blood, and MRI Biomarkers in Skogholt’s Disease—A Rare Neurodegenerative Disease in a Norwegian Kindred

**DOI:** 10.3390/brainsci13111511

**Published:** 2023-10-26

**Authors:** Klaus Thanke Aspli, Jan O. Aaseth, Trygve Holmøy, Kaj Blennow, Henrik Zetterberg, Bjørn-Eivind Kirsebom, Tormod Fladby, Per Selnes

**Affiliations:** 1 Department of Neurology, Innlandet Hospital Trust, 2381 Lillehammer, Norway; klaus.aspli@sykehuset-innlandet.no; 2 Institute of Clinical Medicine, University of Oslo, 0316 Oslo, Norway; trygve.holmoy@medisin.uio.no (T.H.); bjorn-eivind.kirsebom@unn.no (B.-E.K.); tormod.fladby@medisin.uio.no (T.F.); 3 Research Department, Innlandet Hospital Trust, 2381 Brumunddal, Norway; jan.aaseth@inn.no; 4 Department of Neurology, Akershus University Hospital, 1478 Nordbyhagen, Norway; 5 Institute of Neuroscience and Physiology, Department of Psychiatry and Neurochemistry, The Sahlgrenska Academy at the University of Gothenburg, 43153 Mölndal, Sweden; kaj.blennow@neuro.gu.se (K.B.); henrik.zetterberg@clinchem.gu.se (H.Z.); 6 Clinical Neurochemistry Laboratory, Sahlgrenska University Hospital, 43153 Mölndal, Sweden; 7 Institut du Cerveau et de la Moelle Épinière (ICM), Pitié-Salpêtrière Hospital, Sorbonne Université, 75651 Paris, France; 8 First Affiliated Hospital of USTC, University of Science and Technology of China, Hefei 230001, China; 9 Department of Neurodegenerative Disease, UCL Institute of Neurology, Queen Square, London WC1N 3BG, UK; 10 UK Dementia Research Institute, University College London, London WC1N 3AR, UK; 11 Hong Kong Center for Neurodegenerative Diseases, Hong Kong, China; 12 Wisconsin Alzheimer’s Disease Research Center, University of Wisconsin School of Medicine and Public Health, University of Wisconsin-Madison, Madison, WI 53706, USA; 13 Department of Neurology, University Hospital of North Norway, 9019 Tromsø, Norway; 14 Department of Psychology, Faculty of Health Sciences, UiT, the Arctic University of Norway, 9019 Tromsø, Norway; 15 Department of Research, Akershus University Hospital, 1478 Nordbyhagen, Norway

**Keywords:** tau protein, amyloid beta, PDGFRβ, β-trace protein, NFL, GFAP, blood–brain barrier, Skogholt’s disease, MRI

## Abstract

Skogholt’s disease is a rare neurological disorder that is only observed in a small Norwegian kindred. It typically manifests in adulthood with uncharacteristic neurological symptoms from both the peripheral and central nervous systems. The etiology of the observed cerebral white matter lesions and peripheral myelin pathology is unclear. Increased cerebrospinal fluid (CSF) concentrations of protein have been confirmed, and recently, very high concentrations of CSF total and phosphorylated tau have been detected in Skogholt patients. The symptoms and observed biomarker changes in Skogholt’s disease are largely nonspecific, and further studies are necessary to elucidate the disease mechanisms. Here, we report the results of neurochemical analyses of plasma and CSF, as well as results from the morphometric segmentation of cerebral magnetic resonance imaging. We analyzed the biomarkers Aβ_1––42_, Aβ_1–40_, Aβ_x–38_, Aβ_x–40_, Aβ_x–42_, total and phosphorylated tau, glial fibrillary acidic protein, neurofilament light chain, platelet-derived growth factor receptor beta, and beta-trace protein. All analyzed CSF biomarkers, except neurofilament light chain and Aβ_1/x–42_, were increased several-fold. In blood, none of these biomarkers were significantly different between the Skogholt and control groups. MRI volumetric segmentation revealed decreases in the ventricular, white matter, and choroid plexus volumes in the Skogholt group, with an accompanying increase in white matter lesions. The cortical thickness and subcortical gray matter volumes were increased in the Skogholt group. Pathophysiological changes resulting from choroidal dysfunction and/or abnormal CSF turnover, which may cause the increases in CSF protein and brain biomarker levels, are discussed.

## 1. Introduction

Skogholt’s disease is a rare, slowly progressing, central and peripheral neurodegenerative disorder only observed with maternal inheritance in a small Norwegian family line. So far, it has been diagnosed in four generations in a community in the southeastern part of Norway, and it was named after the local community physician who first described it [1,2]. Minor symptoms may develop before the age of 30, but clinical symptoms typically present between the ages of 30 and 70 in affected family members and include cognitive decline, progressive muscle weakness, unsteady gait, and dysarthria [2]. The disorder was first described in 1998 by Hagen and co-workers [2], who reported the clinical characteristics and the results of laboratory, imaging, and nerve conduction studies, as well as histopathological results and a preliminary genetic workup.

They found greatly elevated cerebrospinal fluid (CSF) concentrations of total protein and extensive cerebral white matter lesions, together with signs of peripheral nervous system involvement, originally interpreted as a combined central and peripheral demyelinating disorder [2]. In three of four selected cases examined using electroencephalography (EEG), there were signs of general slowing. In three sural nerve biopsies from selected cases, they found demyelination with teased fibers, great variation in myelin thickness, paranodal globules, onion-bulb formations, and Pi granules but no axonal degeneration. Neurography showed reduced motor conduction velocities and prolonged distal latencies in the peroneal or tibial nerves in three cases. The needle electromyography of two cases showed signs of peripheral neurogenic lesions predominantly in the lower extremities. Agarose gel electrophoresis of CSF showed what were described as transudative patterns (increased protein concentrations) but few or no traces of intrathecal IgG synthesis [2].

Later findings included greatly increased concentrations in the CSF of copper (Cu), iron (Fe), total tau (t-tau), and phosphorylated tau (p-tau), combined with normal to low levels of amyloid beta 42 protein (Aβ_42_) [3,4], but normal CSF cell counts and no signs of intrathecal synthesis of immunoglobulins [3]. 

Subjectively experienced cognitive difficulties are common, and a cognitive screening battery disclosed a significantly prolonged Trail-Making-Test-B time, suggesting some difficulty in executive functioning [3].

The cause of the strikingly unusual combination of clinical and paraclinical findings in Skogholt’s disease is unknown but likely represents a genetically linked neurodegenerative condition not described outside the affected family line [1,2,3,4].

In the present study, our objective was to further study the pathophysiology of Skogholt’s disease, as well as to disclose the characteristics of this disease. We applied a comprehensive battery of markers in CSF and blood, including the core markers of Alzheimer’s disease (AD), specifically the amyloid-β species Aβ_x–42_, Aβ_x–4o_, and Aβ_x–38_; phosphorylated tau (p-tau); and total tau (t-tau), as well as the astroglia-associated glial fibrillary acidic protein (GFAP), the axonal biomarker neurofilament light chain (NfL), the microvascular marker platelet-derived growth factor receptor beta (PDGFRβ), and beta-trace protein (βTP). In addition, we obtained segmental data of the cortical thicknesses and volumes of brain substructures using algorithmic processing of brain MRI data. 

## 2. Materials and Methods

### 2.1. Subjects

We included three separate groups: Skogholt cases, lab controls, and MRI controls. Table 1 shows the demographic data of each group.

### 2.2. Diagnostic Criteria

Belonging to the affected family line was a prerequisite to consider a diagnosis of Skogholt’s disease. An additional increase in CSF total protein above 1 g/L (normal range: 0.15–0.45 g/L) was considered sufficient for the diagnosis. Increased CSF total protein in the range between 0.45 and 1.0 g/L was also considered sufficient for a definite diagnosis if accompanied by clinical symptoms or MRI findings consistent with Skogholt’s disease.

### 2.3. Skogholt Group

Eleven Skogholt patients that were capable of informed consent and physically fit enough to attend were included in the study. All cases belonged to one kindred from a community in the southeastern inland part of Norway. Three additional cases were unable to participate due to age-related frailty or concurrent morbidity at the time of inclusion.

### 2.4. Laboratory Controls

The lab control group consisted of 14 individuals recruited from the Department of Neurology at the Innlandet Hospital Trust in Lillehammer. Patients not expected to receive an inflammatory or neurodegenerative diagnosis were targeted for inclusion, while patients with certain multiple sclerosis or known dementia were not considered eligible. The lab control group was thus heterogeneous, as previously described [3]. Samples of blood and CSF were obtained from the lab controls and compared with the Skogholt group.

### 2.5. Cerebral MRI Controls

For the comparison of cortical and parenchymal segmentation data, we included 60 MRI controls from the Dementia Disease Initiation (DDI) project, which has more than 500 participants [5]. We only included healthy controls, i.e., individuals without biomarker evidence of cerebral amyloidosis (based either on a negative flutemetamol-PET scan or a CSF Aβ_42/40_ ratio ≤ 0.077), with normal results on a cognitive screening battery, and without subjective cognitive complaints. Two eligible controls were excluded due to failed algorithmic parcellation of MRI data.

### 2.6. Ethics

The study protocol was approved by the Regional Committee for Medical and Health Research Ethics, South-East Region, Norway, Ref. No. 556-04224 and No. 2013/1017. The study was conducted in accordance with the Declaration of Helsinki. Written informed consent was obtained from all patients before enrollment.

### 2.7. Lab Pre-Analytics

Samples of plasma and CSF were obtained and prepared as described previously [3]. Before collection, CSF opening pressure was measured. Skogholt patients were sampled in the morning while fasting. A fasting regimen was not feasible for the controls, who were sampled as soon as possible upon inclusion.

### 2.8. Lab Analytics

From CSF and EDTA-plasma samples obtained previously [3], we analyzed an expanded set of brain biomarkers at the Clinical Neurochemistry Lab in Mölndal. CSF total and phosphorylated tau (T-tau and P-tau) as well as Aβ_1–42_ and Aβ_1–40_ concentrations were measured using a fully automated Lumipulse instrument (Fujirebio, Ghent, Belgium) as described previously [6]. The Aβ species Aβ_x–38_, Aβ_x–40_, and Aβ_x–42_ were measured using a MesoScale Discovery triplex assay. CSF NfL and GFAP concentrations were measured using in-house ELISAs as previously described [7,8,9]. Soluble PDGF-receptor β (PDGFRβ) was measured using a PDGFR beta Human ELISA Kit (Thermo Scientific, Frederick, MD, USA).

The beta-trace protein (prostaglandin D synthase) concentration was measured via nephelometry on an Atellica NEPH 630 System (Siemens Healthineers, Erlangen, Germany). 

The measurements were performed by board-certified laboratory technicians who were blinded to the clinical data.

### 2.9. MRI Systems, Sequence Parameters, and Software for Postprocessing Statistics

Cerebral MRI of Skogholt patients was obtained on a Philips Achieva MRI system with a magnetic field strength of 1.5 Tesla. The MRI protocol included 3D FLAIR, axial T2, diffusion, inflow angio, and SWI as well as 3DT1 scanning before and after contrast enhancement with a macrocyclic gadolinium contrast agent (gadoteric acid). 

MRI from the DDI controls used in the current study were obtained on eight scanners distributed in six centers. The scanner systems, sequence parameters, and number of subjects are detailed in Table S1. 

Cortical reconstruction and volumetric segmentation were performed with FastSurfer [10,11]. This included the segmentation of the subcortical white matter (WM) and deep gray matter structures and the parcellation of the cortical surface [12,13] according to a previously published parcellation scheme [14]. This labeled the cortical sulci and gyri, and mean thickness values were calculated in the regions of interest (ROIs). All segmentations were visually inspected.

White matter hyperintensities [15] (WMHs) were segmented from 3D FLAIR MRI using an in-house deep learning algorithm [16].

### 2.10. Statistics

All statistical computing and data visualizations were performed in RStudio using R version 4.1.3 (and 4.2.2 for the final graphs) [17] with extension packages [18,19,20,21,22].

Due to the small numbers of cases and controls, judgements regarding the distribution of data were unreliable. Therefore, we used both Student’s two-tailed two-sample t-test and the Wilcoxon rank-sum test, i.e., the Mann–Whitney U-test, with α pragmatically set to 0.01 when judging group differences and correspondingly provide the raw data of descriptive statistics for each group using the mean and median with the standard deviation (SD) and interquartile, i.e., 1st to 3rd quartile, range (IQR). Group differences in standardized measurements or standardized ratios (measurements with ICV for MRI volume markers) are explored in graphs showing the standardized linear regression coefficients (with 95% confidence intervals) of group status for each marker.

The adjustment of coefficients was carried out by including appropriate covariates in the regression models. Continuous variables such as age and ICV were standardized, while dichotomous variables, e.g., sex or MRI magnetic field strength (1.5 or 3T), were kept unchanged as binary variables.

To verify the confidence intervals, they are also shown as the 2.5th to 97.5th percentile ranges of the bootstrap distributions of the estimates/coefficients based on 20,000 resampled datapoints in the dataset stratified by group and gender.

## 3. Results

### 3.1. Demographics

The Demographics of the Skogholt cases and the two control groups are given in Table 1.

### 3.2. CSF Biomarkers

The opening pressure upon lumbar puncture was normal in all Skogholt patients, ranging from 10.5 to 19.5 cm water, with a mean of 14.4 cm, which was not significantly different from the controls.

The measured CSF biomarkers were significantly increased in the Skogholt group (*t*-test and Mann–Whitney U-test), except for Aβ_1–42_, Aβ_x–42_, and NfL, as shown in Table 2 and Figure S1. (Aβ_1–42_, Aβ_x–42_, and NfL were borderline significantly increased according to the U-test but not the *t*-test).

All directly measured CSF markers except NfL were significantly increased in proportion to the CSF total protein content. The Aβ_1–42/−1–40_ ratio was decreased with borderline significance.

### 3.3. Plasma Biomarkers

Only plasma GFAP was decreased with borderline significance in the Skogholt group, while all other examined plasma biomarkers were not significantly different between the groups (plasma NFL was decreased with borderline significance according to the U-test but not the *t*-test) (Table 3).

### 3.4. MRI Findings

**Figure 1:**
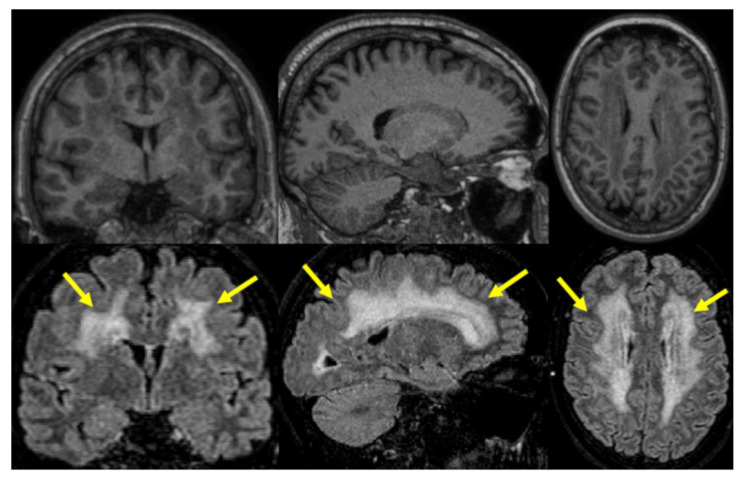
Representative cerebral MRI-T1 (top) and MRI-FLAIR (below) images from a 50-year-old Skogholt patient. Note the extensive white matter hyperintensities (WMHs) typical of the disease (see arrows).

The majority of the examined Skogholt cases had grade-three Fazekas, i.e., confluent deep white matter T2 hyperintensities on cerebral MRI (Table 4 and Figure 1), while most controls had minimal to no observable deep white matter lesions [23]. The observed white matter lesions were not like the typical lesions observed in multiple sclerosis and were considered nonspecific regarding etiology.

**Figure 2:**
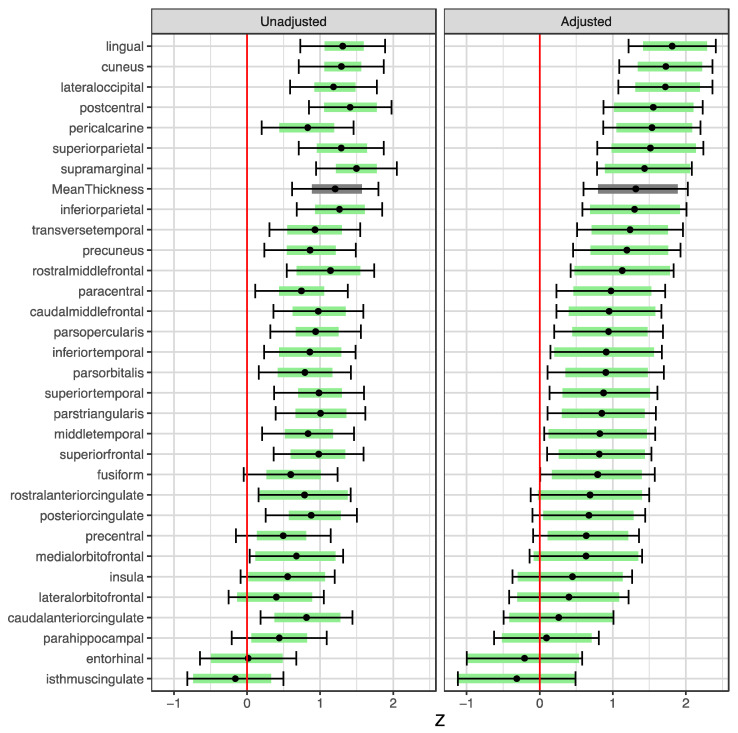
**Differences in regional cortical thickness between the Skogholt subjects and controls.** Group differences in unadjusted standard scores (left pane) and standard scores adjusted for covariates (right pane) of thicknesses of cortical segments on MRI. Dots show estimated differences in standard scores between Skogholt patients and MRI controls. Error bars indicate the 95% CIs, and shaded areas represent the 2.5–97.5 percentile ranges of the bootstrap distributions of the estimated differences from 20,000 resampled datapoints. The standard scores were adjusted for intracranial volume, age, sex, and MRI magnetic field strength using linear regression. Green indicates a particular cortical area, while gray indicates the cortex in general.

The intracranial volume (ICV) was significantly smaller in the Skogholt group, even when adjusting for age, sex, and magnetic field strength (Figure 2). Only a few Skogholt patients had more than minimal cerebral cortical atrophy (Table 5). Two cases had minor microhemorrhages on their SWI sequences, and one additional case had changes in their SWI and diffusion sequences, interpreted as a subacute stroke.

**Figure 3:**
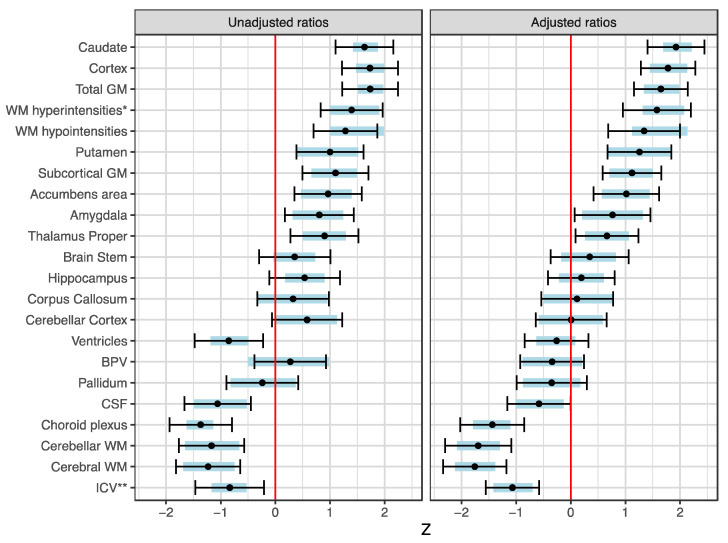
**Differences in brain structure volumes between the Skogholt subjects and controls.** Group differences in unadjusted standardized ratios (left pane) and standardized ratios adjusted for covariates (right pane) of brain segments to intracranial volume (ICV), based on MRI segmentation data from 3D T1 sequences. GM = gray matter, WM = white matter, WMH* = WM hyperintensities on 3D FLAIR sequence. Dots show standard scores for the differences between Skogholt patients and MRI controls. Error bars indicate the 95% CIs, and shaded areas represent the 2.5th to 97.5th percentile ranges of the bootstrap distributions of the estimated differences from 20,000 resampled datapoints. The differences in standardized ratios were adjusted for age, sex, and MRI magnetic field strength using a linear regression with standardization of continuous variables. ICV** = ICV with linear model using standardized ICV value rather than standardized ICV/ICV ratio.

An algorithmic evaluation of the MRI scans showed generally increased cortical thickness in the Skogholt group, even when adjusting for age, gender, magnetic field strength, and ICV (Figure 3). The cortical thickening was most prominent in the lingual, cuneus, and lateral occipital regions.

Relative to the ICV (and adjusted for age, gender, and magnetic field strength) and compared to healthy controls from the DDI project, the Skogholt cases had reduced total white matter, CSF, and choroid plexus volumes and increased white matter changes (Figure 3). The volumes of cortical and subcortical gray matter were modestly increased in the Skogholt group.

Summary statistics of the raw data from MRI parcellation are provided in Supplementary Tables S2 and S3.

## 4. Discussion

Increased CSF total protein concentrations, extensive white matter hyperintensities, and pathological CSF biomarkers of neurodegeneration are established characteristics of Skogholt’s disease [2,3,4].

The present work adds to the literature on Skogholt’s disease with an expanded set of CSF and plasma biomarkers and with MRI morphometry. MRI data revealed strikingly decreased white matter volumes, decreased choroid plexus volumes, increased gray matter volumes, and increased cortical thickness compared to the control group. The extended biomarker panel confirmed previous findings of increased CSF T-tau and P-tau [3] and revealed increased CSF levels of several other biomarkers. In contrast, we did not find increased levels of the same markers in plasma. Such exceptionally high levels of CSF biomarkers cannot be explained by any differences in lifestyle risk factors (Table 1).

Although the previously reported low levels of CSF Aβ_42_ in Skogholt’s disease [3] appear to be inconsistent with the higher levels found in the current analysis, this discrepancy may be explained by the even higher levels of Aβ_x–40_ and Aβ_x–38_ found in Skogholt CSF in this study. The ELISA technique (Innotest kit) previously used to measure the Aβ_42_ concentration [3] is susceptible to interference from Aβ_40_ or Aβ_38_ [24]. The current analysis included two different assays for the quantification of Aβ species: a Lumipulse instrument for Aβ_1–40_ and Aβ_1–42_ and a triplex method for Aβ_x–38_, Aβ_x–40_, and Aβ_x–42_.

The CSF beta-trace protein (βTP) was increased almost seven-fold, substantially more than what can be accounted for by the, on average, 3.5-fold increase in CSF total protein previously reported [3]. Increased βTP concentrations stand in contrast to the low mean choroid plexus volume, as the “brain” isoform of βTP mostly originates in choroid epithelial cells [25]. However, the possibly deficient CSF production seems to primarily affect the watery phase of the liquor. Nevertheless, the speciation of the βTP isoforms in CSF and plasma in comparison to other groups may be of interest in future studies. It is notable that other CSF markers were also increased (Table 2 and Figure S1).

High CSF protein concentrations have previously been reported in conditions with slow CSF turnover [26,27]. It is possible that many of the observed protein changes in Skogholt CSF may be explained by perturbed CSF dynamics, reflecting impaired CSF production. An impaired blood–cerebrospinal fluid barrier with a failing dilution of CSF constituents would also increase the concentration of CNS-derived proteins. An intriguing observation was that the CSF levels of the brain biomarkers were markedly increased, from 2–3-fold (for Aβ_38_, Aβ_40_, NfL, and GFAP) up to 8–10-fold (for t-tau and p-tau). The lack of brain atrophy on MRI indicates that these increases are not related to neurodegeneration. A possible explanation is deranged CSF production affecting the dilution and turnover of constituents, which would allow for a higher CSF concentration of brain proteins. Future research should specifically determine the CSF production and clearance rates in Skogholt’s disease. With MRI, we find that the choroid plexus is smaller in Skogholt’s disease compared to controls, a possible clue to the primary pathology of the condition. CSF protein concentrations are traditionally used as a surrogate marker for the blood–brain barrier (BBB) as well as blood–nerve barrier (BNB) integrity. In chronic inflammatory polyneuropathy, which appears to exist in some patients with Skogholt’s disease [2], a disruption of these barriers is thought to cause the clinical symptoms, resulting in an elevation of the CSF protein level. Damage to the blood–CSF barrier causes altered CSF flow rates, modulating the CSF protein content [28]. BNB damage causes an influx of serum proteins into the CSF [29]. Based on present and previous observations, it is tempting to suggest that Skogholt’s disease is precipitated by primary defects in the BBB and BNB. However, normal levels of tau species in plasma argue against the degeneration of the BBB with leakage (as seen in, e.g., cerebral small vessel disease), and alternative explanations related to the hampered production or release of CSF cannot be excluded.

The MRI findings in the Skogholt cases were unexpected. Compared to the controls, we report decreased white matter and increased gray matter volumes. Additionally, we report decreased choroid plexus and ventricle volumes. The decreased white matter volume may be explained by demyelination or degeneration secondary to the pathological composition of the CSF [30]. Previous studies have found that BBB disruption is linked with white matter degeneration [31,32], again strengthening the hypothesis that BBB dysfunction is a primary or central feature of Skogholt’s disease.

Increased gray matter volumes may be explained by swelling or alternatively by compensatory mechanisms, e.g., an increased number of local interneuronal connections and dysmyelination. Dysmyelination and BBB integrity in Skogholt’s disease are topics for future studies.

The scope of the current study is limited by its small numbers of cases and controls available for fluid marker comparison. The lab control group was diverse and was not sampled as early in the morning as was possible for the Skogholt group. The MRI control group was sufficiently large, but lab results were not available for comparison. Furthermore, the cases and the MRI controls were not examined using the same MRI scanner, introducing the possibility for biased scanner results. However, the morphometric differences were consistent for the different MRI systems, and we corrected for field strength. Additionally, if there were differences due to scanner effects, we would not expect to see the present pattern of group differences. Morphometry on 3T systems is known to report increased cortical thickness compared to 1.5 T systems [33], the opposite of what we found in the present publication.

## 5. Conclusions

The extraordinarily high CSF biomarker levels in Skogholt’s disease are remarkable but must be considered together with the overall increase in CSF proteins, which are altogether more compatible with disturbed CSF dynamics than neurodegeneration. MRI findings of confluent white matter lesions are typical but not necessary to establish a diagnosis of Skogholt’s disease. Our algorithmic processing of MRI data showed decreases in white matter and choroid plexus volumes accompanied by increases in cortical thickness and volume, as well as an increased volume of subcortical gray matter.

Future studies on Skogholt’s disease will benefit from including more cases and avoiding systematic biases when the laboratory analysis and neuroimaging are not distributed over several locations.

## Figures and Tables

**Table 1:** Demographics of study groups.

Characteristic	Skogholt (*n* = 11)	Lab Control (*n* = 14)	MRI Control (*n* = 60)
Sex: Female	6 (55%)	11 (79%)	29 (48%)
Male	5 (45%)	3 (21%)	31 (52%)
Age (Yrs)	57 (45, 67)	64 (56, 70)	64 (58, 68)
Coffee (Cups/d)	3.0 (2.2, 4.2)	2.5 (<1, 4.0)	-
Smoking (pkgYrs) ^a^	9 (4, 29)	1 (<1, 22)	-
Alcohol (U/m) ^b^	9 (3, 14)	3 (1, 6)	-
Exercise (H/w) ^c^	≥3 (1–2, ≥3)	1–2 (1–2, ≥3)	-
Education (Yrs)	9 (8, 11)	12 (12, 16)	14 (12, 16)

Summary statistics presented for sex are given by numbers, with groupwise percentages in parentheses; otherwise, data are given as the medians along with the 1st and 3rd quartiles. ^a^ A pack year (pkgYrs) equals smoking 20 cigarettes daily for one year. ^b^ Alcohol consumption in Norwegian alcohol units per month. ^c^ Hours per week.

**Table 2:** CSF biomarkers.

	Mean (SD)		Median (IQR)		*p* -Values		Ratio of	
Analyte	Skogholt (*n* = 7)	Control (*n* = 11)	Skogholt (*n* = 7)	Control (*n* = 11)	t ^a^	U ^b^	Means ^c^	Medians ^d^
Aβ_1– 42_	1687 (1214)	922 (341)	1464 (950–1718)	921 (721–1068)	0.15	0.079	1.83	1.59
Aβ_1–40_	35,385 (7596)	10,868 (2936)	38,528 (29,796–40,531)	10,614 (8788–12,940)	<0.001	<0.001	3.26	3.63
p-Tau	424 (87.1)	44.2 (29.3)	464 (346–476)	36.5 (27.2–49.5)	<0.001	<0.001	9.61	12.7
t-Tau	3147 (467)	400 (250)	3160 (2814–3562)	342 (236–397)	<0.001	<0.001	7.87	9.23
Aβ_1–42/1–40_	0.050 (0.0277)	0.0842 (0.0194)	0.042 (0.034–0.0485)	0.091 (0.0885–0.0963)	0.017	0.022	0.60	0.46
GFAP	51,497 (10,622)	16,677 (7867)	52,373 (45,109–56,718)	16,974 (9830–22,089)	<0.001	<0.001	3.09	3.09
NfL	9138 (11,486)	4738 (9626)	4210 (2851–8818)	1200 (881–4027)	0.403	0.046	1.93	3.51
PDGFRβ	1915 (283)	422 (118)	1936 (1752–2051)	410 (316–523)	<0.001	<0.001	4.53	4.72
βTP	112 (9.41)	16.6 (3.69)	108 (107–120)	16 (15–18.8)	<0.001	<0.001	6.75	6.75
Aβ_x–38_	5497 (504)	1812 (631)	5359 (5211–5829)	1628 (1370–2302)	<0.001	<0.001	3.03	3.29
Aβ_x–40_	12,319 (2361)	4428 (1097)	13,106 (10,829–14,016)	4326 (3820–4901)	<0.001	<0.001	2.78	3.03
Aβ_x–42_	619 (407)	331 (142)	558 (340–663)	307 (266–361)	0.113	0.031	1.87	1.82

Clinical neurochemistry results from analysis of various species of amyloid beta (Aβ) and results from analysis of total tau protein (t-Tau), phosphorylated tau protein (p-Tau), glial fibrillary acidic protein (GFAP), neurofilament light chain (NfL), platelet-derived growth factor receptor beta (PDGFRβ), and beta-trace protein (βTP). All measurements are in pg/mL except for βTP (mg/mL). ^a^ *p*-values from Student’s *t*-test, not adjusted for multiple testing. ^b^ *p*-values from Mann–Whitney U-tests with continuity correction, not adjusted for multiple testing. ^c^ Ratio of means between Skogholt and control patients. ^d^ Ratio of medians between Skogholt and control patients.

**Table 3:** Plasma biomarkers.

	Mean (SD)		Median (IQR)		*p*-Values		Ratio of	
Analyte	Skogholt (*n* = 11)	Control (*n* = 14)	Skogholt (*n* = 11)	Control (*n* = 14)	t ^a^	U ^b^	Means ^c^	Medians ^d^
tTau	38.8 (24.6)	49 (40.8)	33.2 (28.7–39.2)	39.3 (24.6–47.7)	0.447	0.536	0.791	0.844
GFAP	58.8 (27.8)	128 (107)	56.8 (35–81.2)	82 (68.6–153)	0.034	0.033	0.459	0.693
NfL	22.5 (33.7)	83.3 (186)	9.49 (8.46–15.7)	30.1 (14.3–61.2)	0.251	0.025	0.270	0.316
Aβ_40_	97.6 (20.1)	108 (28.1)	92 (89–101)	94.6 (89.2–123)	0.306	0.647	0.906	0.973
Aβ_42_	6.66 (0.9)	6.95 (1.28)	6.47 (6.18–7.03)	6.97 (6.3–7.3)	0.514	0.501	0.958	0.928
pTau181	7.74 (3.24)	8.23 (4.71)	7.33 (5.92–9.34)	5.85 (5.45–10.8)	0.760	0.687	0.940	1.250
Aβ_42_/Aβ_40_	0.0693 (0.00801)	0.0668 (0.0132)	0.0705 (0.063–0.0761)	0.069 (0.0626–0.0766)	0.566	0.851	1.040	1.020

Clinical neurochemistry results: amyloid beta (Aβ), total tau protein (tTau), phosphorylated tau protein 181 (pTau181), glial fibrillary acidic protein (GFAP), and neurofilament light chain (NfL). All measurements are in pg/mL. ^a^ *p*-values from Students *t*-test, not adjusted for multiple testing. ^b^ *p*-values from Mann–Whitney U-tests with continuity correction, not adjusted for multiple testing. ^c^ Ratio of means between Skogholt and control patients. ^d^ Ratio of medians between Skogholt and control patients.

**Table 4:** Fazekas scores of white matter lesions.

Fazekas Score	Skogholt Group *n* = 11	MRI Control Group *n* = 60
0	2 (18%)	13 (23%)
1	1 (9.1%)	36 (64%)
2	2 (18%)	7 (12%)
3	6 (55%)	0 (0%)
missing	0	4

Semiquantitative scores of white matter hyperintensities on cerebral MRI T2 sequences. Statistics presented as n (%). Fisher’s exact test gives a *p*-value < 0.001.

**Table 5:** MRI cerebral atrophy scores.

Score	GCA	MTA	Koedam
**0**	6 (55%)	6 (55%)	5 (45%)
**1**	3 (27%)	5 (45%)	5 (45%)
**2**	2 (18%)	0 (0%)	1 (9.1%)

Atrophy scores from cerebral MRI of eleven cases with Skogholt’s disease. Statistics presented as n (%). GCA = Global Cortical Atrophy, MTA = Medial Temporal lobe Atrophy, Koedam = Posterior atrophy.

## Data Availability

The data presented in this study are available on request from the corresponding author. The data are not publicly available due to restrictions in consent.

## Supplementary Materials

The following supporting information can be downloaded at https://www.mdpi.com/article/10.3390/brainsci13111511/s1, Table S1: MRI scanner system parameters and number of scans; Table S2: Cortical thickness; Table S3: Region of interest volumes; Figure S1: Violin plots with individual CSF biomarker datapoints.
