# Supplementary material for: CSF, Blood, and MRI Biomarkers in Skogholt’s Disease—A Rare Neurodegenerative Disease in a Norwegian Kindred
Source: Brain Sci. 2023 Oct 26;13(11):1511. doi: 10.3390/brainsci13111511 (PMC10669496; doi:10.3390/brainsci13111511)
Supplement: Supplementary file 1 [file brainsci-13-01511-s001.zip › brainsci-2684703-supplementary.pdf]

SUPPLEMENTARY MATERIAL

Table S1: MRI scanner system parameters and number of scans

**Scanner system**	**3D T1-weighted sequence parameters**	**3D FLAIR sequence parameters**	**Number of patients scanned**
Siemens Avanto 1.5T (Siemens Medical Solutions, Erlangen, Germany)	Magnetization-prepared rapid gradient–echo sequence<br>TR/TE/TI = 1700/2.42/1000 ms; FA = 15°<br>matrix = 256 × 256; 144 slices<br>slice thickness = 1.2 mm<br>in-plane resolution = 1 mm^2	TR/TE/TI = 6000/363/2200 ms<br>slice thickness = 1.2 mm<br>in-plane resolution = 1.2 mm × 1.2 mm	10<br>3
Siemens Skyra 3T (Siemens Medical Solutions, Erlangen, Germany)	Magnetization-prepared rapid gradient–echo sequence<br>TR/TE/TI = 2300/2.98/900 ms; FA = 9°<br>matrix = 256 × 256; 176 slices<br>slice thickness = 1.2 mm<br>in-plane resolution = 1 mm^2	TR/TE/TI = 5000/394/1800 ms<br>slice thickness = 1.2 mm<br>in-plane resolution = 1 mm^2	3
Philips Ingenia 3T (Philips Medical Systems, Best, the Netherlands)	Turbo field echo sequence<br>TR/TE/TI = 4.7/2.3/853ms; FA = 8°<br>matrix= 256 × 256; 184 slices,<br>slice thickness = 2.0 mm<br>in-plane resolution = 1 mm^2	TR/TE/TI = 4800/320/1650 ms<br>slice thickness = 2.0mm<br>in-plane resolution = 1 mm^2	10
Philips Achieva 1.5T (Philips Medical Systems, Best, the Netherlands)	Fast Field Echo<br>TR/TE/TI = 7.2ms / 3.3ms / without SPIR; FA = 8°<br>matrix = 256 × 232; 160 slices<br>slice thickness = 1 mm<br>in-plane resolution = 1 mm^2	TR/TE/TI = 4800/313/1660 ms<br>slice thickness = 1.52 mm<br>in-plane resolution = 1.10 mm^2	11
Philips Achieva 3T (Philips Medical Systems, Best, the Netherlands)	Turbo field echo sequence<br>TR/TE/TI = 6.7/3.1/853 ms; FA = 8°<br>matrix = 256 × 256; 170 slices<br>slice thickness = 1.2 mm<br>in-plane resolution = 1 mm^2	TR/TE/TI= 8000/418/2400ms<br>slice thickness=1.2 mm<br>in-plane resolution = 1 mm^2	17
Philips Ingenia 1.5T (Philips Medical Systems, Best, the Netherlands)	Turbo field echo sequence<br>TR/TE/TI = 7.55/3.43/930 ms; FA = 8°<br>matrix = 256 × 256; 157 slices<br>slice thickness = 1.0 mm<br>in-plane resolution = 1 mm^2	TR/TE/TI = 4800/356/1660 ms<br>slice thickness=1.2mm<br>in-plane resolution = 1 mm^2	9
GE Optima 1.5 T (GE Healthcare, Chicago, IL)	Fast spoiled gradient–echo sequence<br>TR/TE/TI = 11.3/5.04/500 ms; FA = 10°<br>matrix = 256 × 256; 156 slices<br>slice thickness = 1.2 mm<br>in-plane resolution = 1 mm^2	TR/TE/TI = 6000/135.6/1840 ms<br>slice thickness=1.2mm<br>in-plane resolution = 1 mm^2	6
Siemens Prisma 3.0T	Magnetization-prepared rapid gradient–echo sequence	TR/TE/TI = 2200/1.47/900 ms; FA = 8°<br>matrix = 256 × 256; 320 slices<br>slice thickness = 1 mm	2

	in-plane resolution = 1 mm^2

Locations: Oslo University Hospital (OUS); Stord Hospital; Aleris Helse, Oslo, private health enterprise; Innlandet Hospital Trust (Lillehammer); Stavanger university hospital (SUS); University Hospital of North Norway (UNN); Haraldsplass Diaconal Hospital (HDS)

Table S2: Cortical thickness. Cerebral MRI measurements of cortical thickness (mm).

Cortical region of interest	**Skogholt** *n = 11*	**MRI-Control** *n = 60*	*p*-value
Supramarginal	2.52 (2.43, 2.57)	2.27 (2.14, 2.35)	<0.001
Lingual	2.08 (2.04, 2.17)	1.91 (1.81, 1.97)	<0.001
Cuneus	1.95 (1.92, 1.99)	1.79 (1.65, 1.84)	<0.001
Superior parietal	2.15 (2.06, 2.18)	1.94 (1.83, 1.99)	<0.001
Postcentral	2.05 (2.00, 2.12)	1.83 (1.74, 1.89)	<0.001
Mean cortical thickness	2.40 (2.36, 2.48)	2.24 (2.17, 2.32)	<0.001
Inferior parietal	2.40 (2.32, 2.47)	2.22 (2.10, 2.30)	<0.001
Lateral occipital	2.18 (2.12, 2.24)	1.99 (1.90, 2.11)	<0.001
Superior temporal	2.83 (2.75, 2.87)	2.67 (2.52, 2.75)	<0.001
Rostral middle frontal	2.29 (2.23, 2.39)	2.12 (1.98, 2.21)	<0.001
Caudal middle frontal	2.41 (2.32, 2.47)	2.24 (2.07, 2.33)	0.001
Pars triangularis	2.34 (2.30, 2.39)	2.16 (2.05, 2.29)	0.001
Posterior cingulate	2.46 (2.35, 2.50)	2.29 (2.21, 2.37)	0.001
Pars opercularis	2.48 (2.41, 2.52)	2.30 (2.19, 2.43)	0.001
Superior frontal	2.50 (2.43, 2.56)	2.35 (2.20, 2.43)	0.002
Middle temporal	2.74 (2.69, 2.75)	2.61 (2.49, 2.67)	0.002
Precuneus	2.33 (2.29, 2.35)	2.20 (2.09, 2.27)	0.002
Transverse temporal	2.34 (2.29, 2.46)	2.10 (1.96, 2.29)	0.003
Paracentral	2.29 (2.22, 2.36)	2.15 (2.04, 2.27)	0.004
Inferior temporal	2.76 (2.71, 2.87)	2.65 (2.52, 2.76)	0.007
Pars orbitalis	2.58 (2.48, 2.62)	2.38 (2.24, 2.53)	0.009
Caudal anterior cingulate	2.49 (2.40, 2.61)	2.33 (2.23, 2.49)	0.011
Pericalcarine	1.64 (1.52, 1.69)	1.52 (1.38, 1.60)	0.014
Medial orbitofrontal	2.44 (2.29, 2.50)	2.29 (2.20, 2.40)	0.027
Fusiform	2.68 (2.57, 2.74)	2.57 (2.48, 2.66)	0.031
Rostral anterior cingulate	2.70 (2.64, 2.96)	2.60 (2.42, 2.73)	0.038
Parahippocampal	2.88 (2.78, 2.92)	2.78 (2.58, 2.90)	0.11
Insula	3.01 (2.95, 3.12)	2.93 (2.79, 3.04)	0.13
Precentral	2.37 (2.27, 2.42)	2.26 (2.09, 2.39)	0.14
Lateral orbitofrontal	2.45 (2.43, 2.55)	2.42 (2.29, 2.51)	0.2
Isthmus cingulate	2.17 (2.11, 2.31)	2.26 (2.16, 2.35)	0.3
Entorhinal	3.41 (3.26, 3.60)	3.46 (3.26, 3.64)	0.8

Statistics presented: Median (IQR) with *p*-values from Wilcoxon rank sum tests.

Table S3: Region of interest volumes. Cerebral MRI volumetry

Structure	Skogholt<br>*n = 11*	MRI-Control<br>*n = 60*	*p*-value
Choroid-plexus (ml)	0.91 (0.80, 1.04)	1.51 (1.28, 1.95)	<0.001
Cerebellum-White-Matter (ml)	22.6 (17.7, 25.9)	29.0 (25.9, 32.1)	<0.001
CerebralWhiteMatter (ml)	382 (326, 455)	493 (459, 530)	0.001
Ventricles (ml)	17 (13, 22)	32 (19, 42)	0.001
Caudate (ml)	7.64 (7.21, 8.09)	6.56 (6.00, 7.15)	0.001
White matter hypointensities (ml)	8.53 (2.23, 10.09)	1.71 (1.33, 2.80)	0.002
White matter hyperintensities (ml)	13.7 (3.3, 17.2)	2.3 (1.0, 4.7)*	0.002
Surface holes (µl)	96 (48, 126)	35 (17, 79)	0.009
ICV (L)	1.37 (1.31, 1.55)	1.57 (1.42, 1.70)	0.01
Pallidum (ml)	3.14 (2.98, 3.83)	3.81 (3.44, 4.16)	0.03
VentralDC (ml)	7.44 (6.64, 8.46)	8.23 (7.67, 8.92)	0.044
Brain stem (ml)	20.45 (19.03, 22.26)	21.88 (20.04, 23.67)	0.13
Cortex (ml)	437 (422, 490)	418 (393, 448)	0.13
Accumbens-area (ml)	1.19 (0.99, 1.32)	1.04 (0.92, 1.22)	0.2
Cerebellum Cortex (ml)	96 (92, 114)	106 (97, 113)	0.4
Thalamus Proper (ml)	12.34 (11.39, 14.12)	13.01 (11.86, 14.49)	0.4
Putamen (ml)	9.01 (8.54, 10.40)	9.00 (8.35, 9.74)	0.4
Corpus Callosum (ml)	3.62 (3.29, 4.16)	3.88 (3.41, 4.26)	0.5
Total Gray matter (ml)	596 (558, 664)	577 (539, 620)	0.5
Hippocampus (ml)	7.14 (6.96, 8.30)	7.77 (7.23, 8.13)	0.5
Amygdala (ml)	3.32 (2.86, 3.96)	3.40 (3.11, 3.79)	0.6
Subcortical gray matter (ml)	51.6 (49.1, 59.6)	54.7 (50.3, 57.9)	0.7

Statistics presented: Median (IQR) with p-values from Wilcoxon rank sum tests. **n=56*

Figure S1. Violin plots with individual CSF biomarker datapoints

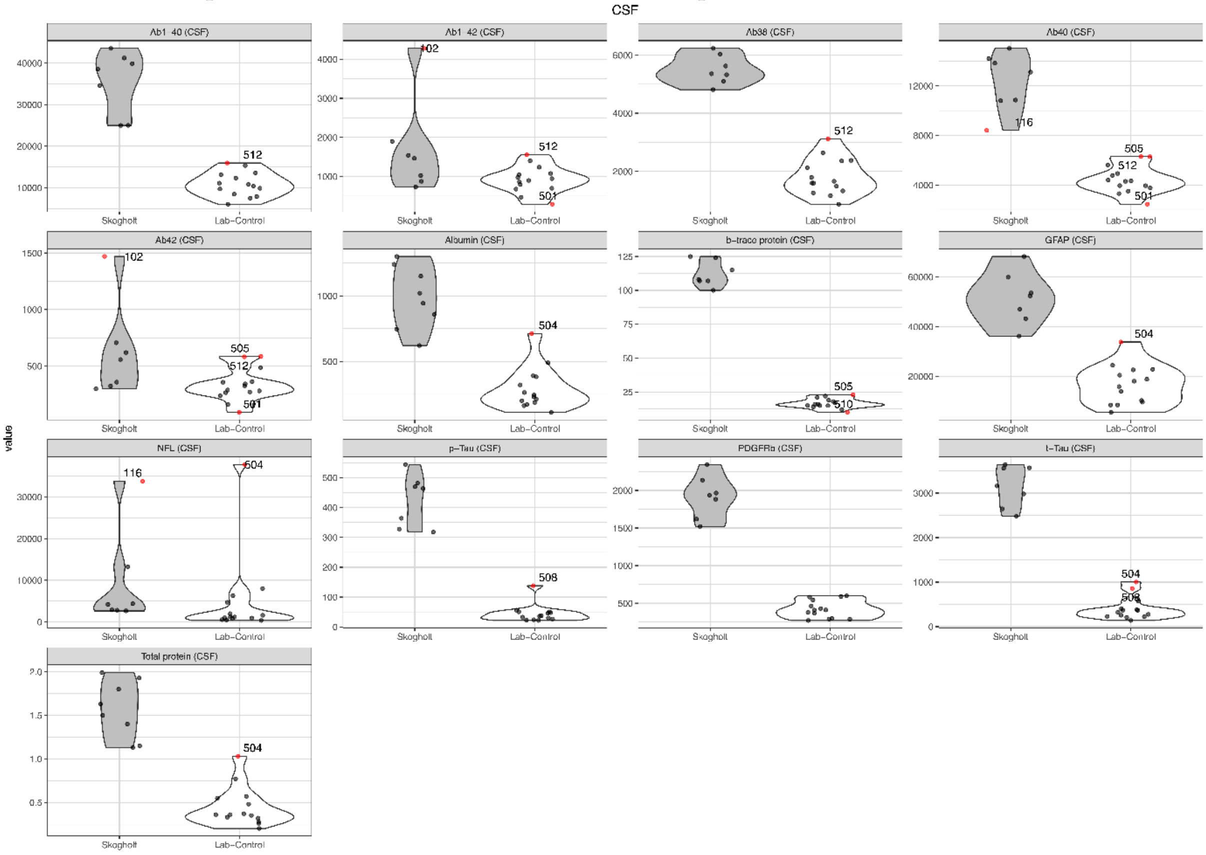

Figure S1. Violin plots with individual datapoints for raw unadjusted values of all measured CSF markers. Red datapoints are extreme values.
